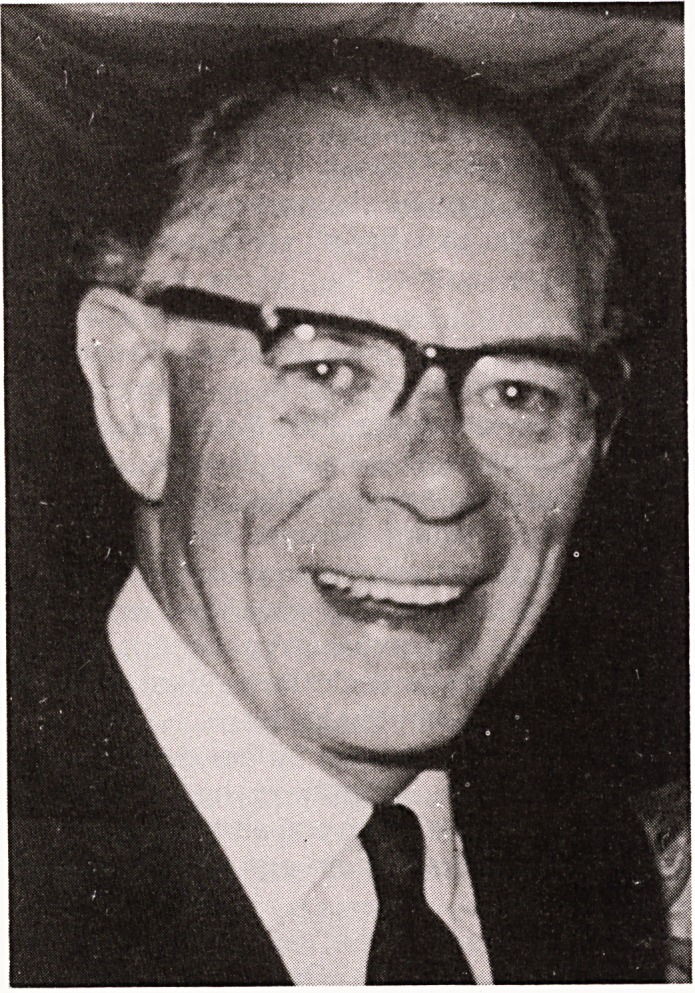# Dr. G. L. Foss

**Published:** 1985-07

**Authors:** 


### Obituary

### Dr. G. L. Foss O.B.E., V.R.D., M.D.

Dr. George Lush Foss who died on 11th February 1985 at the age of 76 years was not only a highly respected General Practitioner in Bristol but also a pioneer in the early days of Endocrinology and the problems of sub-fertility.

Educated at Marlborough and Jesus College, Cambridge he did his clinical training in Bristol where his father was a general practitioner in St. George. On the very day that he graduated in medicine in 1933 he learned that his father had had a stroke and returned to Bristol to perform the evening surgery and take over the practice. In spite of the busy commitments of practice he immediately developed his interest in endocrinology and was Colston Research Fellow from 1935 to 1937 and later honorary assistant in endocrine gynaecology in Bristol. After the war he was appointed adviser in subfertility to the United Bristol Hospitals and medical officer in charge of Bristol's male subfertility clinic. He published altogether 51 papers on a wide variety of aspects of endocrinology and his last paper on the results of AID in collaboration with Mr. Michael Hull was awaiting publication in the *Journal of Obstetrics and Gynaecology* at the time of his death.

George led a very full life. He joined the Royal Naval Volunteers Reserve in 1934 and served for six years in the Royal Navy in the 1939–45 War. He spent three years in carriers in the North Atlantic and Pacific and three in the Chemical Defence Station at Porton and was awarded the OBE He subsequently rose to the rank of Surgeon Captain in the RNVR.

He was a man of wide interests and was for many years a member of Council of Bristol zoo where his knowledge of animal fertility was of great value. He took part in several successful breeding triumphs at the zoo including that of the White Tigers. He was a keen wood carver and clay modeller but his greatest delight and skill was the growing of orchids and he took great pride in the devices of his own design to control the temperature and humidity of his greenhouse. Many a social occasion in Bristol was adorned by his generous gifts of beautiful orchids.

The last fifteen years of George's life were marred by generalised arterial disease which although it restricted his physical activities did nothing to impair his active and enquiring mind. He kept up his many medical and other interests, including membership of the Bristol Medical Reading Society, to the end, and his courage and determination during his last two weeks were an inspiration to all who knew him.

George was very happily married to Eileen who supported him devotedly throughout. They had two daughters and one son who is a Consultant Orthopaedic Surgeon.

A.T.M.R.

## Figures and Tables

**Figure f1:**